# How University Students Assess Their Water Skills

**DOI:** 10.3389/fspor.2022.887216

**Published:** 2022-05-20

**Authors:** Goran Dimitrić, Milorad Jakšić, Filip Sadri, Dorica Šajber, Tanja Kaurin, Nataša Zenić, Elena Tabakova

**Affiliations:** ^1^Faculty of Sport and Physical Education, University of Novi Sad, Novi Sad, Serbia; ^2^Faculty of Sport, University of Ljubljana, Ljubljana, Slovenia; ^3^Faculty of Law and Business Studies dr Lazar Vrkatić, Union University-Belgrade, Novi Sad, Serbia; ^4^Faculty of Kinesiology, University of Split, Split, Croatia; ^5^Institute of Sport and Physical Education, Russian State University of Physical Education, Sports, Youth and Tourism, Moscow, Russia

**Keywords:** water safety, gender comparison, risk assessment, drowning, swimming

## Abstract

The aim of this study was to determine the gender differences between students' actual and perceived water abilities, how respondents assess risk in the described situations, and whether there are gender differences for those situations. The cross-sectional study was conducted on 150 students aged 19–20 years (males, *n* = 88; females, *n* = 62) from the faculty of sport and physical education, University of Novi Sad. Using calculated frequencies and estimates, students' self-assessment and actual measures of their swimming and survival skills and their perceived risk of drowning are described. Based on the results, Mann-Whitney U tests were applied. The differences between independent variables (gender) were analyzed according to dependent measures (water competency). To determine the significance of the relationship between actual and perceived skills, Spearman-rank correlation coefficients were calculated. The results of this study confirmed gender differences between students' actual and perceived water abilities, and that the male and female students had inaccurate perceptions of their own perceived and real water abilities. Both male and female students, with high precision, assessed their ability to swim long distances (rs = 0.601; rs = 0.694) just as female students assessed their ability to float (rs = 0.698). Male students greatly overestimated their backstroke swimming, while female students underestimated their ability to dive into the water. Both groups overestimated underwater swimming and underestimated their surface dive skill. Also, there was gender differences between students in assessing the risk for described situations.

## Introduction

Activities for work or pleasure that are near or on the water have drowning risks. An awareness of this risk, along with achieving water safety knowledge and skills improve enjoyment and safety. When staying near or in the water, it is very important to take care of your own safety and the safety of others. Safety is “a state in which hazards and conditions leading to physical, psychological or material harm are controlled in order to preserve the health and well-being of individuals and the community” (Maurice et al., 2001). When you are confident, it is much easier to enjoy. Perceived motor skills are not real motor skills, but are someone‘s perception of own possibilities (Logan et al., 2015). If someone wrongly perceives their own competencies, especially if they overestimated them, he can be in great danger. An important factor of water competencies is the ability to accurately assess the actual level of one's own skills in water (Stallman et al., 2017). It was previously determined that males (Petrass et al., 2012) and children (Queiroga et al., 2013) could approximately predict their own water skills. Some research has shown that males cannot predict their swimming abilities correctly in moving water (Kjendlie et al., 2013), in swimming in clothes (Moran, 2015), and in safely getting out of the water (Moran, 2014). It should be emphasized that younger children more often overestimate their real abilities in the water (Costa et al., 2020). It can be especially dangerous for young children if their parents also overestimate their abilities (D'Hondt et al., 2021). Drowning can occur anywhere there is water: oceans, seas, lakes, pools, bathtubs, rivers, or even water collections on the side of the road. Drowning is the 3rd leading cause of unintentional injury and death worldwide, accounting for 7% of all injury-related deaths (World Health Organization., 2021). Among many factors influencing adult fatal and non-fatal drowning, mortality is lacking a level of risk awareness, knowledge on water safety (Wu et al., 2017), an overestimation of their own self-assessed swimming skills (Moran et al., 2012), and a lack of water competencies (Stanley and Moran, 2016).

Awareness of the risk of drowning is a signpost on how to spend time next to or in the water without negative consequences. Insufficiency of knowledge and practice on prevention of drowning influence insufficient awareness of the risk of drowning among children (Farizan et al., 2021). Many countries are developing plans to reduce drowning. Drowning and failed rescue attempts have resulted in physiological, sociological, legal, and financial consequences for the injured, the rescuer, and society (Avramidis, 2009). Reducing the number of drownings can be achieved by implementing prevention measures in parallel and raising awareness of drowning, which has been implemented by high-income countries (HIC). This measure has been successfully applied in both low- and middle-income countries (LMICs) (World Health Organization., 2014). Adults and parents should be especially aware of the risks for young people and children. In public swimming pools where there is a high risk of drowning, the absence of risk awareness is best seen (Brenner et al., 2003). It was found that younger men are less likely to improve their safety even though they are aware of the risks of drowning (Titchener et al., 2011). Supervision is mandatory for children under the age of 6 without swimming abilities. Related authorities can organize public cardiopulmonary resuscitation (CPR) classes, establish strict regulations on safety at the pool, and raise community awareness about the risk factors of drowning (Jeswani et al., 2021). Risk-awareness is a way to change safety culture (Hopkins, 2005). Applying this definition to staying in the water, the assessment of the risk of drowning implies considering the situation and making a decision on further activities based on one's abilities.

When and where there is a risk of drowning or injury, water safety invokes the precaution, procedures, and policies that correlate with the safety of the bodies in and on the water or around it (US Coast Guard Auxiliary, 2022). Some of the strategies of HIC for the prevention of drowning are as follows: raising awareness, educating the public, and increasing supervision (Ramos et al., 2015). In these countries, as in others, adults should have good water skills as they are responsible for supervising children around the water where there is a possibility that they are closest to the scene of the incident and have to react (Peden et al., 2019). Water safety education is constantly advancing and improving, so this process is defined as a dynamic process “in which effectiveness is the result of multi-level interaction between the individual, the environment, and the task at hand” (Langendorfer, 2015). Water safety education trains people to acquire knowledge of recognizing and reducing the potential risk in aquatic environments and water activities. The program also includes personal survival and water rescue skills which can help them save themselves in the event of an incident in the water (Red Cross., 2014). There are also somewhat broader definitions of water skills, which say that water safety education is like any other education. The goal of this education “is about equipping people of all ages with the right skills, knowledge, and experience to make informed decisions about their own safety and protect themselves and possibly others from situations that may harm them” (RoSPA., 2008). Although research suggests a positive link between water safety education and the reduction of drowning (Red Cross., 2014), it should be noted that increasing knowledge or awareness alone does not necessarily change safety behavior (Wright, 2016).

There is a self-assessment model which starts from the assumption to render future outcomes more predictable and controllable. People seek to assess abilities that determine important future outcomes (Trope, 1980). This interpretation indicates the need for a good assessment of one's own abilities for being safer next to and in the water. Self-assessment of ability is very important because research indicates that adult men, children, and their caregivers often underestimate water incidents (Morrongiello et al., 2013). Other studies reported that men are more likely to overestimate more water abilities than women (Moran and Stanley, 2013; Rejman et al., 2020). Studies analyzing the personal water competency conclude that most think they swim well, while twice as many men and women think they can swim longer than 200 m and feel safer in open water (Stanley and Moran, 2016). In addition, more men are confident in their rescue abilities (Stanley and Moran, 2018). Also, there is an opinion of younger adults who do not believe that injuries in or on the water can be prevented, and that they can do everything to improve their own safety (Costa et al., 2020). Every swimming training program should ultimately have an assessment of what has been learned based on which each participant or his or her parent would know what his or her abilities are.

Foremost, it is recommended to endorse swimming ability as a necessary module of water skills, but it is also significant to consider the fact that the ability to swim, every so often, is not enough to prevent drowning (Brenner et al., 2006). In the following years, there was an explanation that it is a set of abilities of an individual in the water, which reduce the risk of drowning and increase the ability to perform various tasks in the water (Langendorfer, 2011). Water competence was defined in a drowning prevention context as “the sum of all personal aquatic movements that help prevent drowning as well as the associated water safety knowledge, attitudes, values, judgment and behaviors that facilitate safety in, on and around the water” (Moran, 2013). There is international research that identifies 15 water competencies that include physical, cognitive, and affective characteristics that should prevent drowning (Stallman et al., 2017). These competencies are safe entry (entry, surface, and level off), breath control, stationary surface (front and back float, tread water), water orientation (turn and roll), swimming competencies (on the front, back, and side), underwater competencies (surface dive, swim underwater), use of lifejackets (and other flotation devices), safe exit, clothed water competencies, open water competencies, knowledge (of local hazards and water safety rules), critical decision making–assessing and managing the risk, assess personal competency–to cope with the risk, recognition/assisting a drowning person, and attitudes and behaviors. The importance of floating and swimming, among other water competencies, as a preventive measure against drowning, is emphasized (Stallman et al., 2017; Langendorfer et al., 2018). Nowadays, water competency implies a method to forestall, avoid, and survive drowning situations, along with the ability to foresee, identify, and help persons in possibly dangerous situations. It incorporates water skills, water intelligence, and helping others (Pool Safely collaborator Water Safety USA., 2020).

The objectives of this study are to determine the gender differences between students' actual and perceived water abilities, and also how respondents assess risk in the described situations and whether there are gender differences in risk assessment in the described situations.

## Materials and Methods

Water competencies are one of the drowning prevention measures. Knowing your own abilities in water can be an important factor in water safety for each individual. This is the reason why this study was conducted. The cross-sectional study was conducted on 150 students aged 19–20 years (males, *n* = 88; females, *n* = 62) from the faculty of sport and physical education, University of Novi Sad. The research was conducted according to the protocol of the project “Can you swim?” (Moran et al., 2012). All respondents voluntarily joined the research and signed consent forms when they were acquainted with the purpose of the research and the manner of its implementation. The research consisted of **two** parts. In the **first** part of the research, the respondents filled in a questionnaire which referred to some forms of water abilities and survival skills. The **second** part of the research was testing **six** water skills. This part of the research lasted for **ten** consecutive days wherein 30 students took **three** tests per day. The testing was conducted in a pool that was 25 m long and 2.2 meters deep, with a water temperature of 23°C and a constant presence of lifeguards. Testing was completed before the beginning of the practical part of the lessons in the pool in order to avoid the possible effects of learning from the practical part of the activity. The water skills assessment was conducted **5** days after the end of the survey to avoid completing the survey based on the results achieved in-water skills. The evaluation of real skills in the water, according to the stated standards, was performed by **two** swimming coaches. These skills were as follows: distance swimming, backstroke swimming, floating, dive entry (head first), surface dive (head first), and underwater swim. Swimming skills were assessed by continuous swimming for 15 min using any technique and speed. The swimming distance of each respondent was assessed on a five-point scale: <50 m, 51–100 m, 101–200 m, 201–300 m, and >300 m. Backstroke swimming was assessed by 100 m swim on the back using any technique and speed. This skill was assessed on a four-point scale as follows: did not complete, completed with poor form, completed with satisfactory form, and completed with good/excellent form. To assess respondents' floating skills, they were asked lay on the water surface with minimal movement. This skill was assessed on a four-point scale: <2 min, 2–6 min, 7–15 min, and >15 min. Dive entry skill was tested with dive (head first) into the pool. This skill was assessed on a four-point scale: did not complete, completed with poor form, completed with satisfactory form, and completed with good/excellent form. Then underwater swim was tested. The respondents started with a dive (head first) and swam underwater as far as they could. This skill was assessed on a five-point scale: did not complete, completed 10 m, completed 15 m, completed 20 m, and completed 25 m. Surface dive (head first) was tested with surface dive (head first) to the bottom of the pool. This skill was assessed on a four-point scale: did not complete, completed with poor form, completed with satisfactory form, and completed with good/excellent form. The study was conducted according to the guidelines of the Declaration of Helsinki and was approved by the Institutional Review Board (or Ethics Committee) of the University of Novi Sad, Faculty of sport and physical education, Novi Sad, Serbia (Ref. No. 47-12-11/2021-1).

The collected data were processed by the statistical program IBM SPSS (20.0). Using calculated frequencies and estimates, students' self-assessment and actual measures of their swimming and survival skills and their perceived risk of drowning are described. Shapiro-Wilk test was used to test the normality of the distribution (***p*** <0.001). Based on the results, Mann-Whitney U tests were applied, the differences between independent variables (gender) were analyzed according to dependent measures (water competency). To determine the significance of the relationship between actual and perceived skills, Spearman-rank correlation coefficients were calculated. Spearman's correlation coefficients (r) were used to investigate associations among actual and perceived water skills. The degrees of statistically relevant Spearman's correlation is defined in the relationship as trivial, very small, insubstantial, tiny, practically zero (±0–0.1); small, low, minor (±0.1–0.3); moderate, medium (±0.3–0.5); large, high, major (±0.5–0.7); very large, very high, huge (±0.7–0.9); nearly, practically, or almost; and perfect, distinct, infinite (±0.9–1) (Cohen, 1988). The level of significance was set at ***p*** ≤ 0.05.

## Results

At the beginning of the research, there were a total of 164 students. During testing, some of them did not do a questionnaire or did not complete tests in the water. Incomplete data were excluded, and the research continued with a sample of 150 students. Analyzing the results in Table 1 which shows the students' self-assessed water competencies, it is evident that only 5% of respondents estimated that they could not swim more than 50 m continuously for 15 min using any technique and speed. The assessments of the other respondents were very uniform (22–26%). A large number of the respondents (64%) estimated that they could stay afloat <2 min, and a small number of them (2.7%) could stay afloat longer than 15 min. Analyzing the surface floating ability by gender, it is noticeable that there is a statistically significant difference. Much more male students (76.1%) estimated that they could stay afloat <2 min, and only male respondents (4.5%) could stay afloat longer than 15 min. More female respondents (53.2%) could stay afloat in the range of 2–15 min. Most students estimated that they could swim 100 m on their back (76.7%), dive into the deep end of the pool (90%), can swim underwater (90.7%), and surface dive to a depth of 2 m (85.3%). Statistically, differences were found by gender in self-estimates of water competencies for diving into the pool where all male students estimated that they can dive into the pool and for surface dive to a depth. Meanwhile, a small number (22.6%) of female students estimated that they could not dive to a depth of 2 m.

**Table 1:** Students self-estimated water competencies by gender.

	**Total**		**Male**		**Female**		**Mann-Whitney U**	***p***
	***N***	**%**	***N***	**%**	***N***	**%**		
How many nonstop laps of a 25 m pool can you swim?	
<50 m	8	5.3	3	3.4	5	8.1	2577.000	0.554
51–100 m	39	26.0	28	31.8	11	17.7		
101–200 m	34	22.7	21	23.9	13	21.0		
201–300 m	36	24.0	14	15.9	22	35.5		
>300 m	33	22.0	22	25.0	11	17.7		
How long can you stay afloat?
<2 min	96	64.0	67	76.1	29	46.8	1899.500	<0.001
2–6 min	39	26.0	17	19.3	22	35.5		
7–15 min	11	7.3	0	0	11	17.7		
>15 min	4	2.7	4	4.5	0	0		
Can you swim 100 m on your back?
Yes, can swim 100 m nonstop back	115	76.7	69	78.4	46	74.2	2613.000	0.549
No, c'an't swim 100 m nonstop back	35	23.3	19	21.6	16	25.8		
Can you dive into the deep end of the pool?
Yes, can dive headfirst into the pool	135	90.0	88	100.0	47	75.8	2068.000	<0.001
No, can't dive headfirst into pool	15	10.0	0	0	15	24.2		
Can you swim underwater?				
Yes, can swim underwater	136	90.7	80	90.9	56	90.3	2712.000	0.904
No, can't swim underwater	14	9.3	8	9.1	6	9.7		
Can you surface dive to a depth of 2 m?		
Yes, can surface dive to 2 m	128	85.3	80	90.9	48	77.4	2360.000	0.022
No, can't surface dive to 2 m	22	14.7	8	9.1	14	22.6		
Total	150	100	88	58.7	62	41.3		

According to Table 2, just 29% of students were able to swim nonstop more than 300 m and 4% swam <50 m. Analyzing the swimming ability by gender, there is noticeable statistically significant difference. Most female students (64.5%) were able to swim nonstop more than 200 m compared to male students (21.5%), and fewer female students (8.1%) could not swim for more than 50 m. Most male students (77.3%) swam 50–200 m compared to female students (27.4%). The small number of respondents (13%) showed ability to float. Particularly, female students (27%) floated for 2–6 min compared to men (2%), which is a statistically significant difference. On the backstroke swim test, participants are of similar ability. The students were statistically significantly better at diving into the pool. Students (68%) performed the dive as excellent, while female students (13%) did not dive into the pool. In the underwater swim, participants are of similar ability. Male students (97%) were statistically significantly better in surface dive, while female students (19%) did not complete the task.

**Table 2:** Student water competencies by gender.

	**Total**		**Male**		**Female**		**Mann-Whitney U**	***p***
	***N***	**%**	***N***	**%**	***N***	**%**		
Swimming ability								
<50 m	6	4.0	1	1.1	5	8.1	1743.000	<0.001
51–100 m	40	26.7	32	36.4	8	12.9		
101–200 m	45	30.0	36	40.9	9	14.5		
201–300 m	15	10.0	4	4.5	11	17.7		
>300 m	44	29.3	15	17.0	29	46.8		
Floating ability								
<2 min	131	87.3	86	97.7	45	72.6	2042.000	0.003
2–6 min	19	12.7	2	2.3	17	27.4		
7–15 min	0	0	0	0	0	0		
>15 min	0	0	0	0	0	0		
100 m swim on back								
Did not complete	27	18.0	20	22.7	7	11.3	2513.500	0.388
Completed with poor form	27	18.0	11	12.5	16	25.8		
Completed with satisfactory form	64	42.7	41	46.6	23	37.1		
Completed with good/excellent form	32	21.3	16	18.2	16	25.8		
Dive into pool (2 m depth)
Did not complete	9	6.0	1	1.1	8	12.9	1922.000	<0.001
Completed with poor form	14	9.3	4	4.5	10	16.1		
Completed with satisfactory form	39	26.0	23	26.1	16	25.8		
Completed with good/excellent form	88	58.7	60	68.2	28	45.2		
Underwater Swim								
Did not complete	50	33.3	25	28.4	25	40.3	2331.500	0.118
Completed 10 m	36	24.0	22	25.0	14	22.6		
Completed 15 m	26	17.3	16	18.2	10	16.1		
Completed 20 m	15	10.0	9	10.2	6	9.7		
Completed 25 m	23	15.3	16	18.2	7	11.3		
Surface dive 2 m								
Did not complete	15	10.0	3	3.4	12	19.4	2153.000	0.022
Completed with poor form	41	27.3	25	28.4	16	25.8		
Completed with satisfactory form	42	28.0	25	28.4	17	27.4		
Completed with good/excellent form	52	34.7	35	39.8	17	27.4		
Total	150	100	88	58.7	62	41.3		

If the perceived and real water abilities of male students are analyzed, there is a noticeable (Table 3) high statistically significant relationship between real and expected distance swimming skills. This skill is very important for the safety of the individual in the water. There are also moderate, negative, and statistically significant relations between perceived and actual 100 m backstroke swimming, underwater swimming skills, and surface diving skills. A negative sign of the relations indicates a wrong assessment of skills in the water, which can be very dangerous for any individual. In this sample, the negative sign indicates an overestimation of the backstroke swimming 100 m, underwater swimming skills, and underestimation of the surface diving skills.

**Table 3:** Male students-comparison of estimated and real water competencies.

	**Swim** **estimate**	**Float** **estimate**	**Backstroke estimate**	**Dive** **entry**	**Underwater swim**	**Surfacedive**
				**estimate**	**estimate**	**estimate**
Swim	0.601^**^					
Float		0.085				
Backstroke			−0.421^**^			
Dive entry				-		
Underwater swim					−0.335^**^	
Surface dive						−0.331^**^

** *Correlation is significant at the 0.01 level (2-tailed)*.

By analyzing the perceived and real abilities in the water of the female students, there is a noticeable (Table 4) high statistically significant relationship between perceived and real ability of distance swimming skills and floating skills. As mentioned, these two skills are very important for the safety of the individual in the water. There is a moderate, negative, and statistically significant relationship between actual and expected 100 m backstroke swimming, underwater swimming skills, and surface diving skills. A negative sign of the relations indicates a wrong assessment of skills in the water, which can be very dangerous for any individual. In this sample, a negative sign indicates an overestimation of the underwater swimming skills and an underestimation of surface diving skills.

**Table 4:** Female students-comparison of estimated and real water competencies.

	**Swim** **estimate**	**Float** **estimate**	**Backstroke estimate**	**Dive** **entry**	**Underwater swim**	**Surfacedive**
				**estimate**	**estimate**	**estimate**
Swim	0.694^**^					
Float		0.698^**^				
Backstroke			0.222			
Dive entry				−0.107		
Underwater swim					−0.353^**^	
Surface dive						−0.352^**^

** *Correlation is significant at the 0.01 level (2-tailed)*.

In addition to assessing their water skills, respondents also assessed the degree of risk in the five situations described to them. Analyzing respondents‘ responses (Table 5), it is noticeable that there is no statistically significant difference between the answers of male and female respondents. When asked about the risk assessment when they were caught in the current on a surfing beach, they ran a toy into the deep end of the swimming pool and fell fully clothed into a deep river. In all three described situations, female students were more careful than male students in risk assessment. The existence of a statistically significant difference in the answers can be noticed in the questions for the situation of overturning a canoe 100 m from the shore (*p* = 0.046) and being swept off isolated rocks while fishing (*p* = 0.005). The answers of the female students to these questions indicate their even greater caution compared to male students for the previously described situations.

**Table 5:** Perceptions of risk of drowning by gender.

	**Total**		**Male**		**Female**		**Mann-Whitney U**	***p***
**Risk scenario**	***N***	**%**	***N***	**%**	***N***	**%**		
**Capsized canoe 100 meters offshore**				
Extreme risk	20	13.3	10	11.4	10	16.1	2231.000	0.046^*^
High risk	24	16.0	13	14.8	11	17.7		
Slight risk	46	30.7	23	26.1	23	37.1		
No risk	60	40.0	42	47.7	18	29.0		
**Caught in rip current at surf beach**				
Extreme risk	22	14.7	13	14.8	9	14.5	2532.000	0.435
High risk	44	29.3	22	25.0	22	35.5		
Slight risk	54	36.0	35	39.8	19	30.6		
No risk	30	20.0	18	20.5	12	19.4		
**Chased toy into deep end of swimming pool**				
Extreme risk	6	4.0	3	3.4	3	4.8	2559.000	0.328
High risk	10	6.7	5	5.7	5	8.1		
Slight risk	10	6.7	5	5.7	5	8.1		
No risk	124	82.7	75	85.2	49	79.0		
**Fell into deep river when fully clothed**			
Extreme risk	8	5.3	5	5.7	3	4.8	2579.000	0.530
High risk	15	10.0	9	10.2	6	9.7		
Slight risk	48	32.0	25	28.4	23	37.1		
No risk	79	52.7	49	55.7	30	48.4		
**Swept off isolated rocks while fishing**				
Extreme risk	14	9.3	6	6.8	8	12.9	2038.000	0.005^*^
High risk	24	16.0	10	11.4	14	22.6		
Slight risk	45	30.0	25	28.4	20	32.3		
No risk	67	44.7	47	53.4	20	32.3		
**Total**	150	100	88	58.7	62	41.3		

## Discussion

When analyzing the assessments of abilities of the respondents in distance swimming, there were no big differences between the two groups of respondents, unlike the analysis of skills where female students were significantly better due to how more of them swam over 200 m. This difference between gender in distance swimming could be a consequence of better swimming abilities, physical constitution, and better motivation. Both groups of respondents were highly realistic in assessing their distance swimming ability. The ability to swim for a long time in some incidents can save lives. Swimming ability is one of many ways to prevent drowning (Brenner et al., 2003). Participation in formal swimming lessons is associated with a reduction in the risk of drowning (Brenner et al., 2009).

Floating is the main skill of survival in water and a method of preventing drowning (Andrews, 2019). Both in the assessment and in the real situation, female students were statistically significantly better than male students in the skill of floating on water. All respondents rated their ability to float in water well. The female's rating was highly accurate, and the male rated it well as not being able to swim for long. A smaller number of female students were able to float for <2 min, and more of them floated for 2–6 min. Such difference between gender in floating ability could be a consequence of the physical constitution and the ability to relax while lying on a surface. This ability can be useful after a long swim or a stressful situation in the water; to lay on your back, relax, breathe, concentrate, and decide what to do next. Basic water competency skills, floating, diving, underwater swimming, and swimming technique, are the essence of the concept of water competence and survival skills (Langendorfer and Bruya, 1995; Stallman et al., 2011). There are claims that some different skills from the above are the main skills in the water that may be crucial in a drowning situation. These skills are: buoyancy control (floating), treading water, re-orienting oneself, breath control, and propulsion above and below the water surface (Stallman et al., 2008; Hulteen et al., 2018).

Assessment of swimming ability on the back and real possibilities are similar between two groups of respondents. The male students moderately overestimated their capabilities. This overestimation of the skill of swimming on the back in males is a consequence of their opinion that lying on the back makes breathing “easier.” Hence, they would be able to swim 100 m, which proved to be incorrect. A poor assessment of this ability could jeopardize the safety of an individual who may once have set out to swim a section in this way. It is especially dangerous when abilities are overestimated. Swimming on the backside allows easier breathing, solid propulsion, and poor forward visibility. Drowning survivors who did not know how to float or swim on their backs had to be rescued (Stallman et al., 2008).

In HIC, jumping and diving into water is a small but persistent cause of death and serious injury, especially among male youth and young adults (Moran Dr et al., 2021). Diving headfirst is a popular water activity, but the risk of head, neck, and spinal cord injury means that diving could be extremely dangerous. Diving injuries as a consequence of aquatic recreational activities are the cause of devastating trauma, primarily affecting the cervical spine (Korres et al., 2006). All the male students wrote that they knew to dive headfirst into the pool, with a third of the female respondents saying that they did not know to dive headfirst. In the skill of diving on the head in the pool, the male students were better, and during the testing, more of them were rated as satisfactory and excellent. There are fewer female students than they estimated. Those who did not dive headfirst into the water underestimated their skills. The observed difference between gender in diving into the water in favor of males is caused by their desire to prove themselves and their courage. The headfirst entry in the pool is most often used by divers and swimmers in competitions. Bathers also like to use this type of diving for enjoyment. After such entry into the water, the airways are “closed” and the eyes are in contact with water for some time, which could be a problem for individuals. This skill is important because it indicates that there is no fear of diving into the water, which can sometimes be the only way to enter the water, especially in incidental situations.

According to the underwater swimming skills, respondents are similar in both assessments and tests. However, in their assessment, both groups of respondents significantly moderately overestimated their abilities. This overestimation of underwater swimming skills from a large number of respondents is a consequence of their opinion that they will easily demonstrate this skill, which turned out to be incorrect. The analysis of the real abilities of the respondents shows a somewhat greater ability of males in this skill. In some circumstances, swimming underwater may be a competence required to avoid drowning (Stallman et al., 2017). Moving underwater can be of great benefit to individuals if they find themselves in a situation where their safety or life depends on crossing a certain distance underwater or on their ability to hold their breath for a while.

When analyzing the perceived and tested surface diving abilities, both groups of respondents are quite different. Both groups of respondents moderately underestimated their surface diving skills. This result may be a consequence of their caution in assessing activities that are somewhat more dangerous for them. By analyzing the tested values, males have significantly better results, which are reflected in a smaller number of respondents who could not dive as well as a larger number of respondents who did very well, while the number of girls who did not surface dive is less than estimated. The skill of surface diving can be very useful in incidents in the water, or to get out if there is an obstacle in the water that they cannot otherwise get around or in a situation to help someone (take him out of the bottom of the pool).

When assessing the risk in the described situations, there is a statistically significant difference in “Capsized canoe 100 meters offshore” (0.046) and “Swept off isolated rocks while fishing, situations” (0.005). In both situations, men estimate that there is no risk at all, while girls are more careful in their assessment. These results confirm the WHO report, which states that men drown more often, and the main risk factors include low awareness of water dangers (World Health Organization., 2014).

The results of this study confirmed gender differences between students' actual and perceived water abilities. The results of this research show that the male and female students had inaccurate perceptions of their own perceived and real water abilities. According to perceived abilities, female students were much better at floating and male students were much better at diving into water and surface dive. In real situations, in the water, female students were much better at distance swimming and floating, and men at diving and surface dive. When assessing the risk, there was gender differences between students. The male students claimed that there was no particular risk when a canoe capsized 100 m offshore and if they are swept off an isolated rock while fishing. It is very important that students are aware of their own abilities in the water so as not to endanger their own lives or the lives of others.

## Limitations of the Research

There are several limitations which must be which must be mentioned. The respondents in this research are students of the faculty of sports and physical education who are much more capable than their peers who are from other faculties. The research was cross-sectional, and such research can be organized and have a longitudinal character. Future research could be conducted for students to assess their own swimming skills in clothing, river, or wave swimming skills.

## Data Availability Statement

The raw data supporting the conclusions of this article will be made available by the authors, without undue reservation.

## Ethics Statement

The studies involving human participants were reviewed and approved by University of Novi Sad, Faculty of Sport and Physical Education Ref. No. 47-12-11/2021-1. The patients/participants provided their written informed consent to participate in this study.

## Author Contributions

GD and NZ wrote the article. GD, MJ, TK, FS, DŠ, NZ, and ET designed the study, analyzed the data, discussed the results, reviewed, and approved the article. All authors contributed to the article and approved the submitted version.

## Funding

This work has been supported by the Provincial Secretariat for Higher Education and Scientific Research (142-451-2594).

## Conflict of Interest

The authors declare that the research was conducted in the absence of any commercial or financial relationships that could be construed as a potential conflict of interest. The handling Editor AB declared a past co-authorship with one of the authors NZ.

## Publisher's Note

All claims expressed in this article are solely those of the authors and do not necessarily represent those of their affiliated organizations, or those of the publisher, the editors and the reviewers. Any product that may be evaluated in this article, or claim that may be made by its manufacturer, is not guaranteed or endorsed by the publisher.
